# Training tomorrow’s leaders in global health: impact of the Afya Bora Consortium Fellowship on the careers of its alumni

**DOI:** 10.1186/s12909-016-0750-x

**Published:** 2016-09-19

**Authors:** Aliza Monroe-Wise, Yohana Mashalla, Gabrielle O’Malley, Neal Nathanson, Esther Seloilwe, Onesmus Gachuno, Theresa Odero, Damalie Nakanjako, Nelson Sewankambo, Edith Tarimo, David Urassa, Yukari C. Manabe, Susan Chapman, Joachim G. Voss, Judith Wasserheit, Carey Farquhar, Afya Bora Consortium Working Group

**Affiliations:** 1 Departments of Medicine and Global Health, University of Washington, 325 Ninth Avenue, Box 359909, Seattle, WA 98104-2499 USA; 2 Department of Medicine, University of Botswana, Gaborone, Botswana; 3 Department of Global Health, University of Washington, Seattle, WA USA; 4 Perelman School of Medicine, University of Pennsylvania, Philadelphia, PA USA; 5 School of Nursing, University of Botswana, Gaborone, Botswana; 6 Department of Obstetrics and Gynecology, University of Nairobi, Nairobi, Kenya; 7 School of Nursing Sciences, University of Nairobi, Nairobi, Kenya; 8 Department of Internal Medicine, Makerere University, Kampala, Uganda; 9 College of Health Sciences, Makerere University, Kampala, Uganda; 10 Department of Nursing Management, Muhimbili University of Health and Allied Sciences, Dar es Salaam, Tanzania; 11 Department of Community Health, Muhimbili University of Health and Allied Sciences, Dar es Salaam, Tanzania; 12 Department of Medicine, Johns Hopkins University, Baltimore, MD USA; 13 Department of Social and Behavioral Sciences, School of Nursing, University of California, San Francisco, CA USA; 14 School of Nursing, University of Washington, Seattle, WA USA; 15 Departments of Medicine, Global Health, and Epidemiology, University of Washington, Seattle, WA USA

**Keywords:** Leadership training, Africa, Transformative leadership, Interprofessional, Transprofessional

## Abstract

**Background:**

Effective leadership is a cornerstone of successful healthcare delivery in resource limited settings throughout the world. However, few programs in Africa prepare healthcare professionals with the leadership skills vital to the success of the healthcare systems in which they work. One such program, the Afya Bora Consortium Fellowship in Global Health Leadership, has been training health professionals since 2011. The purpose of this study was to assess what career changes, if any, the Afya Bora Fellowship’s alumni have experienced since completing the fellowship, and to describe those changes.

**Methods:**

The Afya Bora Fellowship is a multidisciplinary, one-year training program that teaches health professionals leadership skills through didactic and experiential learning in four African countries. Between January 2011 and June 2013 the consortium trained 42 nurses and doctors. In November 2013, an electronic survey was sent to all alumni to assess their performance in the workplace post-fellowship.

**Results:**

Thirty-one (74 %) of 42 alumni completed surveys. Twenty-one (68 %) reported changes to their position at work; of those, sixteen (76 %) believed the change was due to participation in the fellowship. All alumni reported improved performance at work, and cited the application of a wide range of fellowship skills, including leadership, research, communication, and mentoring. Twenty-six (84 %) alumni spearheaded improvements in their workplaces and almost all (97 %) remained in contact with colleagues from the fellowship. Among the respondents there were five publications, nine manuscripts in preparation, and three international conference presentations.

**Conclusions:**

Afya Bora alumni overwhelmingly reported that the one year fellowship positively influenced both their work and career trajectory. Training health professionals in leadership skills through didactic modules with the opportunity to apply learned skills at attachment sites in the Afya Bora Fellowship has an impact on performance in the workplace and the potential to improve long-term institutional capacity.

**Electronic supplementary material:**

The online version of this article (doi:10.1186/s12909-016-0750-x) contains supplementary material, which is available to authorized users.

## Background

As the response to the HIV epidemic in Africa matures from an acute to a chronic care model, attention has turned to the role played by the global healthcare workforce and the importance of robust health systems. In addition to the well-documented shortage of health care providers in Africa [1–3], a new focus on the quality and content of health education has emerged recently, highlighting the need for improved training in leadership and management skills [4, 5]. Indeed, leadership has been cited as one of the key ingredients for healthcare reform in low-income settings [6], and the World Health Organization stressed continuing faculty development in its first set of guidelines for transforming and scaling up health professionals’ education and training [7].

Leadership, communication, and vision are valuable attributes for health professionals who ascend to positions in which they design, implement and scale-up health programs and policies. However, the competencies that support these attributes are rarely taught in standard medical or nursing curricula [5, 8]. Competency-based transformative educational programs have been proposed as effective ways to train healthcare leaders to become stronger agents of change in healthcare settings [5]; however, very few such programs exist [9]. Furthermore, while both interprofessional and transprofessional education are vital to the achievement of transformative leadership [5], there is a dearth of programs that currently embody these values in health education worldwide [8, 10], and essentially none for African professionals [10].

According to Knowles’ classic theory of andragogy, adult education is most efficient when learners are self-directed, draw on their personal experiences, feel motivated toward the process of learning, and when the material being learned is immediately relevant to problems faced by the learner [11]. These observations have driven changes in the design of modern medical education programs from didactic to interactive, problem-based learning structures [12]. Furthermore, as adult learning theory underscores the importance of immediate relevance of the material being taught, medical schools increasingly incorporate local examples into case-based discussions [13].

**Fig. 1:**
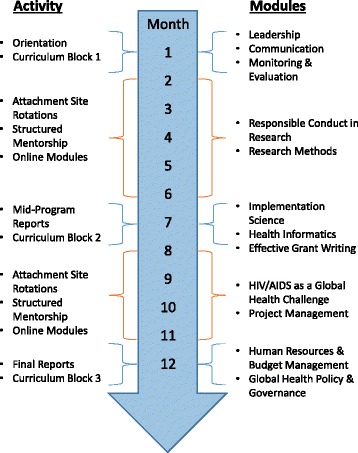
Program theory of change framework for the Afya Bora Fellowship.  =Outcomes of interest for this evaluation

The Afya Bora Consortium Fellowship in Global Health Leadership is a multidisciplinary training program that employs a competency-based curriculum to provide future global health leaders with practical leadership and management skills that are not part of traditional health professional training. Based on the Health Leadership Development Model, the training program was designed to guide fellows through stages of leadership behavior change according to a transtheoretical model. Evaluative data describing leadership behavior change from the pilot year of the fellowship have been published elsewhere [14]. The one year fellowship involves three classroom learning blocks each lasting three weeks, separated by two five month experiential attachment site rotations at local health-related non-governmental and governmental organizations, including the Ministries of Health, in four African countries: Botswana, Kenya, Tanzania and Uganda (Fig. 1). All classroom-based modules employ case-based, small-group discussions and draw on Africa-focused examples to maximize relevance to the learners. Attachment site rotations allow fellows to apply concepts and skills learned in the classroom to real-world problems faced by local health organizations, further maximizing andragogical educational impact. Additionally, this practicum provides learners with a contextual platform on which to test and understand new processes, a key construct in normalization process theory [15].

**Fig. 2:**
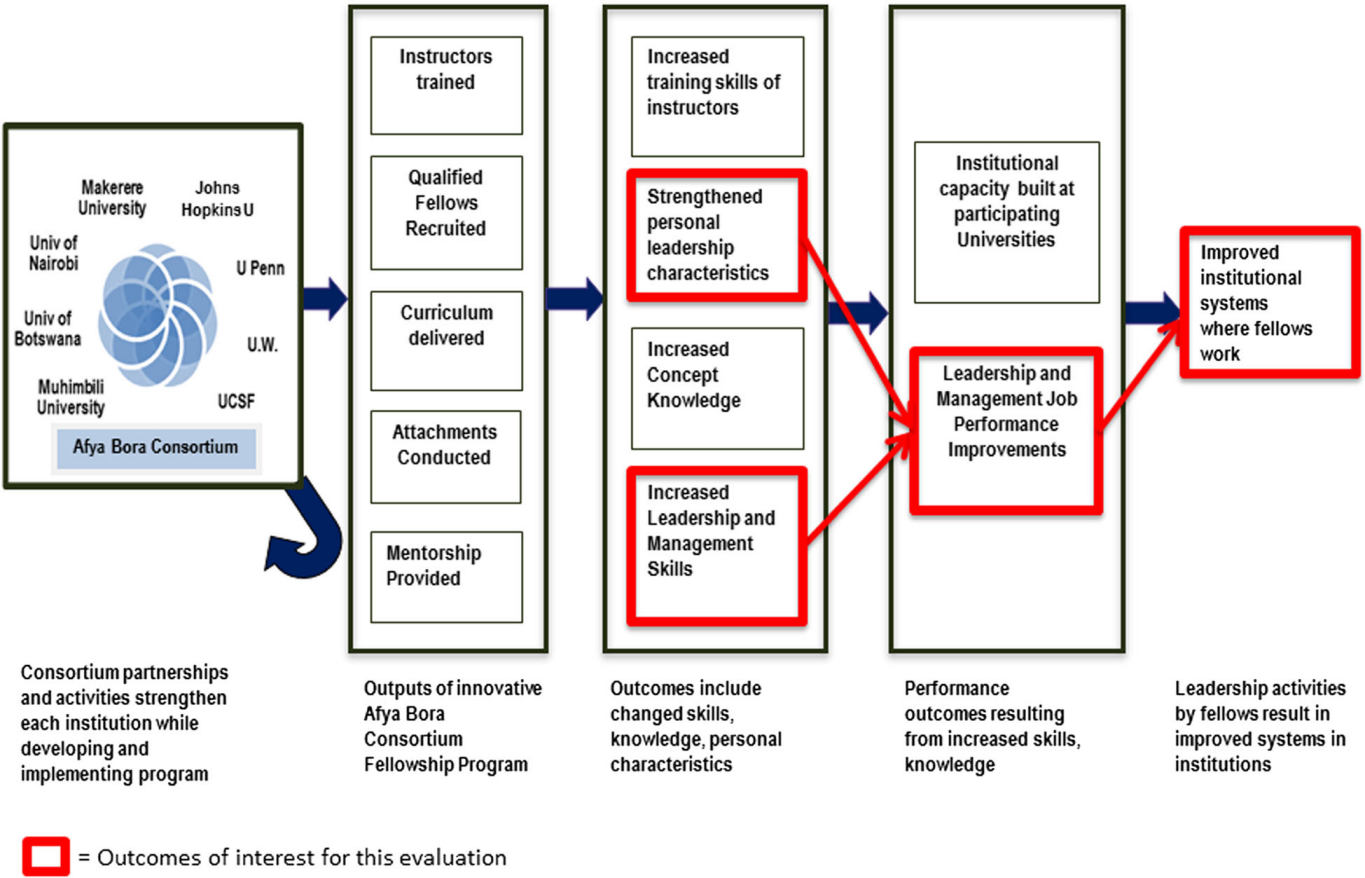
Afya Bora Consortium Fellowship structure and timeline

**Fig. 3:**
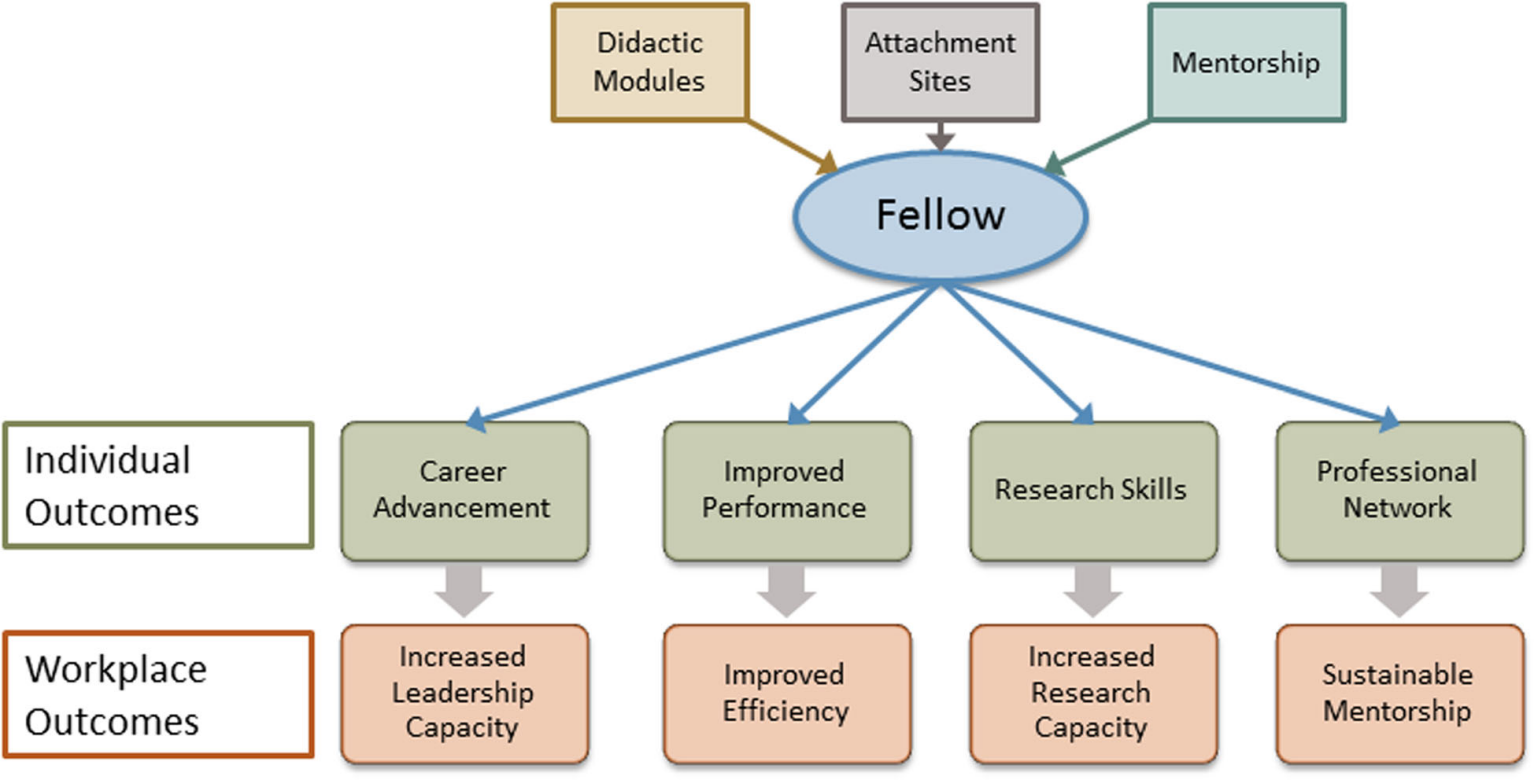
Conceptual map of Afya Bora Fellowship impact

Now in its fourth year, the Afya Bora Fellowship has successfully trained three cohorts of healthcare professionals from Africa and the United States. After learning new skills and concepts through the program, alumni have returned to the workforce in their respective countries and refilled vital positions in the healthcare and education systems. This assessment sought to understand what impact, if any, the fellowship has had on the careers and professional trajectory of its alumni.

## Methods

The Afya Bora Consortium was founded in 2009 based on four pre-existing partnerships between United States (U.S.) and African universities. A pilot fellowship program began in January 2011 and the first full-year fellowship cohort was enrolled in July 2012. A detailed description of the program has been published previously [16]. Since the 2011 pilot of the fellowship program, the consortium has trained two additional cohorts of nurses and doctors from Botswana, Kenya, Tanzania, Uganda, and the U.S.

We used a program theory of change framework [17–19] to guide this 2013 evaluation of the Afya Bora Leadership training program during its third year of operation. Our evaluation was particularly interested in the intermediate performance outcomes and improvements made to health systems (see Fig. 2). In November 2013 an electronic survey was sent to all Afya Bora Fellowship alumni (see Additional file 1). The questionnaire was developed by AMW with input from GO, in order to prompt reflection and reporting on what, if any, effects the Afya Bora experience had on the fellows, and what changes the fellows made in their workplace that they could attribute to the Afya Bora program. Members of the Afya Bora working group reviewed the questions to ensure they were eliciting relevant information based on envisioned outcomes of the program. In order to reduce social desirability bias, the questionnaire asked respondents to provide concrete examples to illustrate responses. Conducted in English, the survey included questions about former and current professional positions, changes to performance at work, relationships with Afya Bora colleagues, ongoing Afya Bora-related activities, and suggestions for enhanced alumni support from the fellowship. Questions were both multiple choice and open-ended paragraph format. Surveys were not sent to alumni from the second year of the program (2013–14) because they had not yet completed the program at the time that the survey was sent. Alumni completed the surveys electronically, and their answers were captured in an online spreadsheet. Although identifying information was included in survey answers, all identifiers were removed prior to data analysis. Participant responses were submitted to the monitoring and evaluation team and no raw data or identifiers were seen by the mentors or program leads of the fellows.

Qualitative data were inductively coded using content analysis [20]. AMW reviewed all of the data and developed an initial set of codes, creating a broad taxonomy of career progression and workplace performance reported by participants, as well as the reported presence or absence of change. She then coded for major themes to further explore dimensions of the taxonomy [21] (see Additional File 2). GO then reviewed all codes and independently coded data. No major discrepancies were identified between text coded by AMW and GO and minor differences in coding were resolved through consensus. Descriptive statistics were compiled in Microsoft Excel.

## Results

Between January 2011 and June 2013, 42 fellows from five countries participated in the Afya Bora Fellowship. This included 22 fellows in the pilot year (January–July 2011), and 20 fellows in the first full year of the program (June 2012-July 2013). Among the 42 fellowship participants in the two cohorts, 25 were women and 17 were men. Eight (19 %) fellows were Batswana, 13 (31 %) Kenyan, 8 (19 %) Tanzanian, 8 (19 %) Ugandan, and 5 (12 %) U.S. citizens. Twenty-one fellows were nurses, and 21 were physicians. The fellows were employed in various health sectors, with 23 coming from academic institutions, 11 from ministries of health or government hospitals, and 8 from non-governmental organizations. Overall, the fellows were highly educated, with 32 holding masters degrees, including masters of public health (MPH), science (MSc), and science of nursing (MSN), eight holding masters’ in medicine (MMed), and six who had completed or were working towards doctoral degrees (PhD).

In November 2013 an electronic survey was sent to all 42 alumni; of these, 31 (74 %) responded. Respondents included 14 (52 %) alumni from the pilot year and 17 (48 %) from the 1st full year (Table 1). Of those who responded, the distribution by country was similar to that of all alumni during this period, with 16 % from Botswana, 32 % from Kenya, 23 % from Tanzania, 16 % from Uganda, and 13 % from the U.S.

**Table 1:** Comparison of all Afya Bora Fellowship alumni to the subset of survey respondents

	All alumni (*N* = 42)		Survey respondents (*N* = 31)	
Pilot Year (2011)	22	(52 %)	14	(45 %)
1^st^ Year (2012–13)	20	(48 %)	17	(55 %)
Botswana	8	(19 %)	5	(16 %)
Kenya	13	(31 %)	10	(32 %)
Tanzania	8	(19 %)	7	(23 %)
Uganda	8	(19 %)	5	(16 %)
United States of America	5	(12 %)	4	(13 %)
Female	25	(60 %)	17	(55 %)
Nurses	21	(50 %)	13	(42 %)
Physicians	21	(50 %)	18	(58 %)

### Career advancement

Twenty-one (68 %) of 31 responding alumni reported that their positions at work had changed since completing the fellowship. Of those who had experienced changes to their position at work, 16 (76 %) believed that the change was due to experience gained through participation in Afya Bora. Promotions or new jobs were the most common types (75 %) of career advancement mentioned. Others had taken on more responsibilities at work, and two had enrolled in higher academic degree programs.

Several program alumni commented on how their training during Afya Bora led to promotions in their workplaces. Specifically, they cited both skills learned during didactic modules and during experiential attachment site blocks, underscoring the importance of both modalities in adult learning theory.

*The leadership and management skills obtained from the modules as well as the project on PMTCT [Prevention of mother-to-child transmission] [that] I undertook during my attachment enabled me to be recognized to fill the position of PMTCT program manager, which was a promotion.*

*-MD30*

Some fellows also stated that after the fellowship, they were in a more competitive position to be considered for new jobs, or felt more confident while applying. This illustrates a separate phenomenon by which the act of completing a leadership training program confers additional qualification for leadership *a priori,* as illustrated here:

*I had been a student for a long time and had not been pursuing leadership/management roles. It was challenging to apply for positions after so long as a “grad student.” Having this type of formal training/experience helped with applications, and then with working.*

*-RN10*

*Also [Afya Bora] did improve on my overall self-presentation, in terms of communication (CV & oral presentation).*

*-RN18*

Other alumni reported achieving higher academic degrees post-fellowship, and using these qualifications to apply for new jobs:

*[Afya Bora] fellowship helped me secure a scholarship for a Masters Training. By [having] a masters, I was able to compete for the current position I am holding.*

*-MD11*

The upward mobility described in these different types of career changes demonstrates the success of the fellowship in training a cadre of professionals on track to become influential leaders in their respective arenas. In their ascent into positions of leadership, fellows were able not only to utilize skills and competencies learned in the fellowship, but also started to incorporate these strengths into the institutional framework of the organizations in which they work, as described below.

### Improved performance

All 31 respondents reported that participation in the program had positively impacted their actions or performance at work. When asked how much Afya Bora had affected their work performance on a scale of 0 to 10, the average response was 8.2 (minimum 5, maximum 10, median 8).

Fellows described a wide range of competencies learned through Afya Bora that were helpful to their work, in addition to a sense of confidence in their own leadership qualities that was not linked to any particular learned skill. Many fellows highlighted changes that utilized their leadership skills, as is evidenced in this quote:

*Before [Afya Bora] I used to think that leading was something very complicated, however after acquiring the leadership skills I felt I was ready to lead. [Afya Bora] improved my qualities as a leader, it also strengthened my monitoring and evaluations skills. I know what to expect as leader and manager. I clearly understand the difference between leadership and management. I am now used as a resource person for project management issues.*

*-RN26*

Others described specific skills they had learned during both didactic modules and attachment site experiential learning. In describing how these new skills affect their work, alumni use language that evidence both action and maintenance phases of behavior change in the Health Leadership Development Model.

*I used many of the skills I learned in the fellowship to plan and develop the training program. From analyzing the problem and systems thinking, to consensus building and stakeholder analysis, I turned to the tools from [Afya Bora] for guidance. Prior to the fellowship, I might not have thought to analyze the situation and gather input from relevant people before developing the program. I might have simply developed a program that I thought would fit their situation, and asked for feedback at the end. Instead, after the fellowship training, I sought input at the beginning and throughout the project. I used this information to inform the development of the program, and I sought frequent consensus on the direction and details of the program. In the end, the program was more relevant for the situation and people involved.*

*-RN02*

Twenty-six (84 %) responding alumni reported that they had spearheaded improvements at their workplaces since completion of the fellowship, evidencing the final stage of leadership behavior change (maintenance stage), in which the leader has acquired a sense of necessary organizational structure change. Many of these involved improved processes or more formal structures to ensure operational efficiency and leadership:

*Afya Bora helped me to get skills to ensure projects do not fail! I was able to demonstrate this in the projects that we do and one key principle I left [Afya Bora] with… is that an absent manager can actually be the reason for a project failure. When I brought this [idea to] some of our organizational meetings it helped to create a system that ensures managers are available and their performances evaluated. I was also able to hire competent staff, including a project manager, coordinators and research assistants and created a mentorship plan for the manager.*

*-RN16*

When applied to the health institutions that alumni work in, the transformative leadership skills mentioned here are likely to improve organizational capacity and efficiency, particularly as alumni advance in the workplace to become higher in leadership structure. As promotions occur, alumni acting as change agents within institutions will have larger effects on the growing areas they oversee.

### Professional network

Almost all (97 %) Afya Bora alumni remained in contact with colleagues from the fellowship. Of these, alumni were most frequently still in contact with other Afya Bora fellows (90 %). Primary mentors were the next most common type of colleague that alumni were in touch with (71 %), followed by attachment site mentors (65 %). Additionally, 21 alumni (70 %) remained directly involved in Afya Bora-related activities, such as ongoing research or other projects with attachment site personnel.

Perhaps more striking than the descriptions of ongoing relationships between alumni and fellowship mentors were the descriptions of how Afya Bora alumni had become mentors themselves in the time since the fellowship. This was a recurrent theme in many of the descriptions alumni provided about their changing positions in the workplace, and is further evidence of transformative leadership. Inherent in these new mentorship roles is a promulgation of leadership qualities that alumni now embody. Additionally, the following quotes describe the process of teaching and transmitting leadership skills that are not typically taught in health workers’ training programs.

*The first thing [Afya Bora] did… was to completely change my communication skills, mainly the scientific writing skills. Based on this I started to mentor doctors to write abstracts [to] send to scientific conferences. All of them (24) were accepted.*

*-RN16*

*I am spending more time with my students [and] interns, coaching & mentoring them & provide info beyond curriculum matter.*

*-RN18*

These examples describe a sustainable ethos of mentorship in the workplace. Further, they provide evidence of the development of interprofessionalism, one of the core tenants of transformative health education. As Afya Bora alumni continue to grow and develop into healthcare leaders in their respective countries, they will bring to the table the emerging understanding that transformative leadership involves supporting and fostering the careers and work of colleagues on different levels.

### Research capacity

Many alumni described different types of success in the research realm, including grant applications, presentations and publications. Among the alumni who responded to the survey there had been five publications, nine manuscripts in preparation, and three conference presentations since completing the fellowship. For many, this success in the academic side of health sciences led to new career directions:

*[Afya Bora] stimulated me to write more research articles in peer reviewed journals, which earned me more publications and helped meet the requirements for promotion.*

*-RN32*

Others found greater success in the academic areas in which they had previously been working:

*Before [my Afya Bora] training we didn’t win competitive grants in other regions of Tanzania apart from Dar es Salaam. But after the [Afya Bora] training we were the only organization that won a grant in [other regions]. We even had some other partners asking us “what did you [do] to get that?” and of course the answer was the capacity of our staff was built by the [Afya Bora] training!*

*[Regarding my] proposal writing skills: I applied those and we [won] a CDC grant to support HIV care program in Kagera region. These were two outstanding performances that my supervisor saw and appointed me to [an] additional 2 positions of website editor and director of a new research project.*

*-RN16*

In addition to describing improved research skills and outcomes, these examples demonstrate the potential that alumni have to contribute to the research capacity both in their workplaces and in their countries. Skills such as proposal, grant, and manuscript writing are useful in a wide range of positions beyond academia, and are vital for the success of many healthcare systems. As Afya Bora alumni continue to rise to influential positions in their workplaces, their training may affect the success of these organizations in competing for programmatic or research grants and other funding opportunities.

## Discussion

While the importance of leadership is widely acknowledged, evaluations of training designed to improve leadership for health are relatively rare in the peer reviewed literature, especially in the context of international global health [19, 22–25]. As such, identifying commonalities between Afya Bora and other leadership programs describing positive outcomes may be helpful to organizations working to improve global health. Shared characteristics across leadership programs include a heavy emphasis on interactive, experiential learning that includes opportunities to practice skills in real world settings, develop professional networks, and meet regularly with mentors [23, 26–28]. With its interactive, case-based didactic curriculum, experiential attachment site rotations rooted in strong mentorship, and cultivation of interprofessional networks through multidisciplinary cohorts, the Afya Bora training program embodies these important principles. Indeed, in describing the professional successes they have enjoyed since completing the fellowship, Afya Bora’s alumni frequently cite skills learned during both didactics and attachment site experiences, multidisciplinary cohorts, mentorship and networking as valuable aspects of the training curriculum.

Designing training programs that take place in-country may reduce the threat of ‘brain drain’ as persons who leave resource-limited settings for higher education programs sometimes do not return to their country of origin [23, 29]. This is not uncommon for leadership training programs for Africans; however, few have cohorts from multiple African countries learning together and creating a South-South (between low-income countries in the Southern hemisphere) network. The duration of global health leadership fellowship programs ranges from 6 [27] to 14 months [23], long enough for trainees to be able to apply principles learned in the classroom setting. The core component of the Afya Bora Fellowship falls within this range at 12 months; furthermore, we have found that alumni continue to interact with each other and their mentors well beyond this period. Finally, to our knowledge, the Afya Bora Fellowship program is the only international leadership program to train cohorts of doctors and nurses together, and to foster South-South networks across these different cadres. This is particularly important in settings with limited human resources for health and promotes interprofessional learning, leading to the development of transformative leadership in multiple disciplines [5].

The interpretation of our evaluation data is limited by several factors. First, our survey was designed to elicit information about changes to and trajectory of the careers of Afya Bora’s alumni. It did not capture any data on specific elements of the fellowship program that may have led to these career changes, and the results may therefore hold limited generalizability and reproducibility. Additionally, it is possible that there was a selection bias in that only those Afya Bora alumni who believed the program had been beneficial responded to the survey. However, if this is the case, it is notable that the response rate was relatively high at 74 %. Also, data were self-reported and identifiable, which has the potential to introduce several biases. However, it should be noted that retrospective self-reported data is a common method of leadership program evaluation [22, 23, 26, 30, 31], and that the alumni have no ongoing professional ties to the evaluators, thus minimizing the potential for social desirability bias.

Finally, Afya Bora participants were highly skilled and motivated before starting the program, and although we ask participants specifically about changes attributable to their Fellowship experience, our data cannot predict whether outcomes similar to those identified by the evaluation would have occurred from the natural course of a highly qualified individual’s career, even without participation in the Afya Bora Fellowship. Regardless, major themes that emerged from the Afya Bora evaluation data resonate with metrics of success used elsewhere, as well as with qualitative research on characteristics of African leadership [24]. These include the capacity to act as a change agent [22, 24, 31]; increased ability to use data for decision making [22, 24]; consensus building through increased communication skills [22, 26, 31]; research and publication [23]; increased capacity to mentor others [22, 31]; career advancement [23]; and organizational performance improvements [22, 23, 27].

## Conclusions

Leadership development is often categorized as a complex, emergent process which requires self-reflection and identification of outcomes and critical incidents leading to outcomes [14, 19,]. In its first two years, the Afya Bora Fellowship has provided leadership training to 42 health professionals from the Botswana, Kenya, Tanzania, Uganda, and the United States. Alumni reported positive impacts of the fellowship on their work, ranging from new positions with more responsibilities to increased expertise, ongoing professional relationships and resulting publications. Our findings (see Fig. 3) indicate that leadership training programs designed to provide interdisciplinary, trans-national experiential learning and focused mentorship to young healthcare professionals in resource poor settings can lead to improved institutional capacity, stronger South-South networks, and, ultimately, an emerging cadre of transformative leaders. Further studies are necessary in order to develop a body of knowledge describing successful African leadership training programs; however, our findings may aid in the development of such programs, as the training model exemplified by Afya Bora is transferrable.

## Additional files

Additional file 1: Afya Bora Alumni Impact Survey: Survey used in this assessment. (CSV 1 kb) Additional file 2: Table S2. Emergent themes from qualitative data analysis. (DOCX 14 kb)

## Abbreviations

MMed: Masters of medicine
MPH: Masters of public health
MSc: Masters of science
MSN: Masters of the science of nursing
PhD: Doctor of philosophy
PMTCT: Prevention of mother-to-child transmission
U.S.: United States of America

## References

[1] Mullan F, Frehywot S, Omaswa F, Buch E, Chen C, Greysen R, Wassermann T, Abubakr DEE, Awases M, Boelen C, Diomande MJMI, Dovlo D, Ferro J, Hailemlak A, Iputo J, Jacobs M, Koumare AK, Mipando M, Monekosso GL, Olapade-Olaopa EO, Rugarabamu P, Sewankambo N, Ross H, Ayas H, Chale SB, Cyprien S, Cohen J, Haile-Mariam T, Hamburger E, Jolley L, Kolars JC, Kombe G, Neusy AJ (2011). Medical schools in sub-Saharan Africa. Lancet.

[2] World Health Organization. Working together for better health. World Health Organization (WHO); 2006. http://www.who.int/whr/2006/en/. Accessed 15 July 2015.

[3] Crisp LN (2011). Global health capacity and workforce development: turning the world upside down. Infect Dis Clin N Am.

[4] World Health Organization. Transformative scale up of health professional education: an effort to increase the numbers of health professionals and to strengthen their impact on population health. World Health Organization (WHO); 2011 http://www.who.int/hrh/resources/transformative_education/en/. Accessed 15 July 2015.

[5] Frenk J, Chen L, Bhutta ZA, Cohen J, Crisp N, Evans T, Fineberg H, Garcia P, Ke Y, Kelley P, Kistnasamy B, Meleis A, Naylor D, Pablos-Mendez A, Reddy S, Scrimshaw S, Sepulveda J, Serwadda D, Zurayk H (2010). Health professionals for a new century: transforming education to strengthen health systems in an interdependent world. Lancet.

[6] Senkubuge F, Modisenyane M, Bishaw T (2014). Strengthening health systems by health sector reforms. Glob Health Action.

[7] World Health Organization. Transforming and scaling up health professionals’ education and training: World Health Organization guidelines; 2013. http://www.who.int/hrh/resources/transf_scaling_hpet/en/. Accessed 15 July 2015. 26042324

[8] Gabel S (2014). Expanding the scope of leadership training in medicine. Acad Med.

[9] Stoller J (2013). Recommendations and remaining questions for health care leadership training programs. Acad Med.

[10] Straus SE, Soobiah C, Levinson W (2013). The impact of leadership training programs on physicians in academic medical centers: a systematic review. Acad Med.

[11] Knowles MS (1970). The modern practice of adult education.

[12] Wood D (2003). ABC of learning and teaching in medicine: Problem based learning. BMJ.

[13] Kaufman D (2003). ABC of learning and teaching in medicine: Applying educational theory in practice. BMJ.

[14] Daniels J, Farquhar C, Nathanson N, Mashalla Y, Petracca F, Desmond M, Green W, Davies L, O’Malley G, Afya Bora Consortium Working Group Members (2014). Training tomorrow’s global health leaders: applying a transtheoretical model to identify behavior change stages within an intervention for health leadership development. Glob Health Promot.

[15] May C (2006). A rational model for assessing and evaluating complex interventions in health care. BMC Health Serv Res.

[16] Farquhar C, Nathanson N (2011). The Afya Bora Consortium: an African-US partnership to train leaders in global health. Infect Dis Clin N Am.

[17] Weiss C (1998). Evaluation Methods for Studying Programs and policies.

[18] Funnell S, Rogers P (2011). Purposeful Programme Theory, Effective use of theories of change and logic models.

[19] Watkins KE, de Marrais K, Lyso IH (2011). Evaluating executive leadership programs: A theory of change approach. Adv Dev Hum Resour.

[20] Patton MQ (2002). Qualitative Research and Evaluation Methods.

[21] Strauss A, Corbin J (1998). Basics of Qualitative Research: Techniques and Procedures for Developing Grounded Theory.

[22] Woltring C, Constantine W, Schwarte L (2003). Does leadership training make a difference? The CDC/UC Public Health Leadership Institute: 1991–1999. J Public Health Manag Practice.

[23] Jones DS, Tshimanga M, Woelk G, Nsubuga P, Sunderland NL, Hader SL, St Louis M (2009). Increasing leadership capacity for HIV/AIDS programmes by strengthening public health epidemiology and management tranining in Zimbabwe. Hum Resour Health.

[24] Curry L, Taylor L, Chen PG, Bradley E (2012). Experiences of leadership in health care in sub-Saharan Africa. Hum Resour Health.

[25] Chen FM, Bauchner H, Burstin H (2004). A call for outcomes research in medical education. Academic Medecine.

[26] Umble K, Baker EL, Diehl SJ, Haws S, Steffen D, Frederick S, Woltring C (2011). An Evaluation of the National Public Health Leadership Institute–1991–2006: Part II. Strengthening Public Health Leadership Networks, Systems, and Infrastructure. J Public Health Management Practice.

[27] Seims LRK, Alegre JC, Murei L, Bragar J, Thatte N, Kibunga P, Cheburet S (2012). Strengthening management and leadership practices to increase health-service delivery in Kenya: an evidence-based approach. Human Res Health.

[28] Chamberlain J, Watt S (2012). Training multidisciplinary leaders for health promotion in developing countries: lessons learned. Health Promot Pract.

[29] Saravia NG, Miranda JF (2004). Plumbing the brain drain. Bull World Health Organ.

[30] Fernandez CS, Noble CC, Jensen E, Steffen D (2015). Moving the needle: a retrospective pre- and post- analysis of improving perceived abilities across 20 leadership skills. Matern Child Health J.

[31] Day M, Shickle D, Smith K, Zakariasen K, Moskol J, Oliver T (2014). Training public health superheroes: five talents for public health leadership. J Public Health.

