# Supplementary material for: Training tomorrow’s leaders in global health: impact of the Afya Bora Consortium Fellowship on the careers of its alumni
Source: BMC Med Educ. 2016 Sep 19;16:241. doi: 10.1186/s12909-016-0750-x (PMC5028919; doi:10.1186/s12909-016-0750-x)
Supplement: Additional file 2: Table S2. — Emergent themes from qualitative data analysis. (DOCX 14 kb) [file 12909_2016_750_MOESM2_ESM.docx]

Table 2. Emergent themes from qualitative data analysis

| **Emergent Theme** | **Illustrative Quote** |
| --- | --- |
| Career Advancement | First, during recruitment the position required [a] candidate with medical background but also education and skills in management. As [an alumnus] of Afya Bora fellowship, I stood on [the] advantageous side compared to my fellow candidates [with] whom [I] shared the medical and technical background but [who] did not have any formal advanced managerial training.  -- MD29  I was promoted because my employer felt that I was now equipped with leadership and management skills.  --RN26  The next level of my promotion will be highly influenced [by] the experience I gained from being an [Afya Bora] fellow.  --RN23  I was hired for this position based on my previous participation in [Afya Bora].  --MD12  I was given more responsibilities with the newly-funded grant compared to my position on the previous grant.  --RN02  I got admitted for Masters of Public Health (MPH) program at Makerere University as soon as I finished [Afya Bora] fellowship, so I have not utilized the knowledge at work yet, but [Afya Bora] helped me so much during my MPH program as some subjects were much related from what we learned during the fellowship.  --MD13 |
| Improved Performance | I used many of the skills I learned in the fellowship to plan and develop the training program. From analyzing the problem and systems thinking, to consensus building and stakeholder analysis, I turned to the tools from [Afya Bora] for guidance.  --RN02  [Afya Bora] impacted on many spheres of my work; to include resource management, communication and conflict resolution strategies.  --RN03  Leadership skills and M&E lectures were so helpful as they have impacted me in the way I do my work. Entirely everything I [learned] from [Afya Bora] was beneficial to me.  --MD04  Specific skills such as drafting plans, budgets and overseeing multiple teams with multiple agendas. Leadership in working as direct supervisor of large, diverse staff.  --RN10 |
| Professional Network | While…I can’t attribute my current position to [Afya Bora], after [Afya Bora] I did work with an NGO… as SRH-Manager where I think [Afya Bora] partly contributed through networking and [the] attachment [site experience], I was able to know about available employment options.  --RN17  I am [a] very good mentor to… students and one tutorial fellow.  --RN06  I have been able to engage with other global teams on HIV and my ability to debate in these meetings is much better. I have been invited as a guest speaker at ICASA to speak on adolescent HIV work in Uganda which we did post- Afya Bora.  --MD07  I do mentor my officers and hold coaching sessions with them.  --RN21 |
| Research Capacity | My knowledge on epidemiology, biostatistics and research methods enabled me to clearly bring out my research question and rationale for my PhD studies.  --RN03  Being involved in a population survey at [the] attachment site gave me a lot of experience in doing research. I have applied for [a] PhD scholarship and I quoted this experience… I hope it will give me an upper- hand in being awarded for scholarship.  --RN06  One of the research projects I'm involved with was a direct result of the things I learned from [Afya Bora].  --MD12  I am able to present papers confidently in National conferences.  --MD20  I have trained other health workers to do research and write manuscripts and grant applications.  --MD05 |
